# A Sense of Being Needed: A Phenomenological Analysis of Hospital-Based Rehabilitation Professionals’ Experiences During the COVID-19 Pandemic

**DOI:** 10.1093/ptj/pzac052

**Published:** 2022-05-05

**Authors:** Roel van Oorsouw, Anke Oerlemans, Emily Klooster, Manon van den Berg, Johanna Kalf, Hester Vermeulen, Maud Graff, Philip van den Wees, Niek Koenders

**Affiliations:** Radboud University Medical Center, Radboud Institute for Health Sciences, Department of Rehabilitation, Nijmegen, the Netherlands; Radboud University Medical Center, Radboud Institute for Health Sciences, IQ Healthcare, Nijmegen, the Netherlands; Radboud University Medical Center, Radboud Institute for Health Sciences, IQ Healthcare, Nijmegen, the Netherlands; Deventer Hospital, Department of Rehabilitation, Deventer, the Netherlands; Radboud University Medical Center, Department of Gastro-Enterology and Hepatology-Dietetics and Intestinal Failure, Nijmegen, the Netherlands; Radboud University Medical Center, Donders Center for Neuroscience, Department of Rehabilitation, Nijmegen, the Netherlands; Radboud University Medical Center, Radboud Institute for Health Sciences, IQ Healthcare, Nijmegen, the Netherlands; HAN University of Applied Sciences, Faculty of Health and Social Studies, Nijmegen, the Netherlands; Radboud University Medical Center, Radboud Institute for Health Sciences, Department of Rehabilitation, Nijmegen, the Netherlands; Radboud University Medical Center, Radboud Institute for Health Sciences, Department of Rehabilitation, Nijmegen, the Netherlands; Radboud University Medical Center, Radboud Institute for Health Sciences, IQ Healthcare, Nijmegen, the Netherlands; Radboud University Medical Center, Radboud Institute for Health Sciences, Department of Rehabilitation, Nijmegen, the Netherlands

**Keywords:** Keywords: Allied Health Care, COVID-19, Experiences, Moral Distress, Professional Autonomy, Qualitative Research

## Abstract

**Objective:**

The purpose of this study was to explore lived experiences of rehabilitation professionals working in hospitals during the COVID-19 pandemic, including the ethical issues and moral distress that these professionals might have encountered.

**Methods:**

An interpretative phenomenological study was performed. First-person experiences of rehabilitation professionals (dieticians, occupational therapists, physical therapists, and speech-language therapists) were collected with semi-structured interviews and analyzed with interpretative phenomenological analysis.

**Results:**

The data of 39 hospital-based rehabilitation professionals revealed 4 themes: a disease with great impact, personal health and safety, staying human in chaotic times, and solidarity and changing roles. Participant experiences show that the virus and COVID-19 measures had a significant impact on the in-hospital working environment due to the massive downscaling of regular care, due to infection prevention measures, and due to unknown risks to rehabilitation professionals’ personal health. At the same time, participants experienced a certain freedom, which made room for authentic motives, connection, and solidarity. Participants felt welcomed and appreciated at the COVID-19 wards and intensive care units and were proud that they were able to fulfill their roles. The findings reflect a wide range of situations that were morally complex and led to moral distress.

**Conclusion:**

To diminish the long-lasting negative impact of the COVID-19 pandemic and moral distress, employers should empathize with the experiences of hospital-based rehabilitation professionals and create conditions for ethical reflection. Our data show that hospital-based rehabilitation professionals value professional autonomy. Creating room for professional autonomy helps them feel needed, connected, and energized. However, the needs of hospital-based rehabilitation professionals may conflict with organizational rules and structures.

**Impact:**

Hospital-based rehabilitation professionals were involved in situations they considered morally undesirable, and they inevitably faced moral distress during the COVID-19 crisis. This study offers rationale and guidance to employers regarding how to reduce the long-term negative impact of the COVID-19 pandemic on rehabilitation professionals.

## Introduction

On March 11, 2020, the World Health Organization declared the outbreak of COVID-19 to be a pandemic. This outbreak, caused by the contagious novel coronavirus (SARS-CoV-2), led to many hospitalizations worldwide.^1^ At the peak of hospitalizations in the Netherlands, in the beginning of April 2020, over 1300 patients with COVID-19 were admitted to intensive care units (ICUs) and over 2600 patients were admitted to hospital wards.^2^

During this first wave, patients hospitalized with suspected or confirmed COVID-19 were cared for, in full isolation and separated from other patients, using “cohorting.” Cohorting allows dedicated staff to work in a ward only with patients who tested positive for COVID-19, so they are unlikely to carry the virus from patients who were positive to patients who were negative. Hospital-based rehabilitation professionals, including dieticians, occupational therapists, physical therapists, and speech-language therapists, were tasked with a variety of roles in the care of patients with COVID-19 in a working environment very different from normal, with limited availability of personal protection equipment (PPE). They had to make decisions with limited knowledge of optimal precaution and treatment strategies. Due to the crisis, the work process was often task based instead of human based and holistic. Although the professional roles were mostly well delineated, rehabilitation professionals could face roles for which they were not prepared.

As earlier outbreaks have shown, decision-making in a time of emergency is associated with ethical issues.^3^ Ethical issues occur when values and norms conflict or when they no longer seem applicable. These conflicts may occur within a person or between different persons, or there may be disagreement with the rules or structures that guide an individual’s professional activities.^4^ In these situations, it is not possible to avoid acting; therefore, decisions must be made.^5^ Making difficult decisions in such troubling situations may result in moral distress.^6^ Different definitions of moral distress are used. For the purposes of this study, moral distress is defined as “1 or more negative self-directed emotions or attitudes that arise in response to one’s perceived involvement in a situation that one perceives to be morally undesirable.”^7^^(p6)^ The core ethical concepts and their operational definitions can be found in Supplementary Material 1. Moral distress among health care professionals can cause professional dissatisfaction, burnout, and shortages in medical staff.^6^

Numerous studies have sought to investigate the lived experiences of health care workers in the COVID-19 pandemic; however, they focused predominantly on physicians and nurses.^8–10^ Few to none of these studies included rehabilitation professionals^11^; however, some included physical therapists.^12^^,^^13^ Insight into their experiences could help to empathize with unusual working situations and identify requirements of rehabilitation professionals during this pandemic or a possible future one. The aim of this study was to explore the lived experiences of rehabilitation professionals working in hospitals during the COVID-19 pandemic, including the ethical issues and moral distress these professionals might have encountered, experienced, or both.

## Methods

### Qualitative Approach and Research Paradigm

We performed an interpretative phenomenological study to explain lived experiences of rehabilitation professionals in 4 hospitals in the Netherlands. Phenomenology is a philosophical approach to the study of experiences developed by the German mathematician Edmund Husserl and those who expanded on his views, such as Heidegger, Sartre, and Merleau-Ponty.^14^ The aim of phenomenological inquiry is to identify the essential qualities of the experience of a particular phenomenon.^15^ It focuses on the appearance of the world to the individual through “going back to the things themselves,” which means that experience should be examined in the way that it occurs, respecting individual differences and suspending judgments about what is real.^15^ In everyday life, our attention is directed outwards to the activities we are engaged in, and we take for granted our experiences.^15^ When we turn inwards and consciously reflect on any of the things we see, think, remember, or wish, then we are being phenomenological and capable of exploring the experiences that we lived through.^15^ We collected first-person accounts of experiences with semi-structured interviews and analyzed the interview data with interpretative phenomenological analysis.^15^ Reporting of this study followed the Standards for Reporting Qualitative Research.^16^ The Radboudumc ethical committee (dossier number 2020–6520) judged that this study does not fall under the scope of the Dutch Medical Research Involving Human Subjects Act (WMO). General principles from the Declaration of Helsinki and Good Clinical Practice were followed.^17^^,^^18^ Written informed consent was obtained from all participants prior to the interview.

### Researcher Characteristics and Reflexivity

The interviews were performed by 3 researchers (R.v.O., E.K., and N.K.) who were all hospital-based physical therapists and involved in health care for patients hospitalized with COVID-19. The interviewers were all educated and experienced in performing qualitative research. Prior to the interviews, the interviewers bracketed their own experiences, opinions, and prejudices on working as a rehabilitation professional in the hospital during the COVID-19 pandemic.^19^ They accomplished this by writing a reflection about their own experiences and the issues they encountered. Before the start of the data analysis, the 2 data analysts (R.v.O. and N.K.) again bracketed their experiences, opinions, and prejudices once more in a reflection report. The other researchers have backgrounds as an ethicist (A.O.), dietician (M.v.d.B.), speech and language therapist (H.K.), nurse (H.V.), occupational therapist (M.G.), and physical therapist (P.v.d.W.).

### Context

The interviews were performed in June and July 2020. During these months, the numbers of hospitalizations concerning COVID-19–infected persons in the Netherlands were low. The lockdown measures in the Netherlands were less restrictive starting May 2020. In June and July 2020, the basic rules still applied: stay home when sick, work from home by default, wash your hands more often than usual, and maintain 1.5 m distance. Public life had somewhat normalized, with students returning to schools, wearing facemasks in public transport, and cafes and restaurants being open with a maximum of 30 persons. No vaccines were available yet during this period.

### Sampling Strategy

Through purposeful sampling, rehabilitation professionals were selected for participation in this study. Optimal variation was sought in sex, age, profession, years of work experience, and type of hospital. Potential participants were eligible for study inclusion if they were employed in a hospital as a dietician, occupational therapist, physical therapist, or speech-language therapist and if they participated in health care for patients hospitalized due to COVID-19 infection. Potential participants were excluded from the study if the professional had no access to digital communication equipment or if the professional suffered from psychological complaints requiring professional support.

### Data Collection

First-person accounts of experiences were collected to study the lived experiences of rehabilitation professionals, setting aside assumptions and prejudices from participants and interviewers about common sense and science. Participants were asked to share their thoughts and experiences in 1 in-depth semi-structured interview of a maximum 60 minutes. The interviews were structured by an interview guide (Suppl. Material 2) covering the following topics: working in the hospital, working with patients with COVID-19, working at the cohort ward, working at the ICU; screening, diagnostics, treatment, ending treatment, and referral of patients with COVID-19; PPE equipment and infection precautions; and ethical dilemmas, moral distress, and personal health. The interviewers stimulated the participants to describe the experiences as they had lived them and to avoid causal explanations, generalizations, or abstract interpretations.^20^ All interviews were held though video calling to avoid the risk of viral spreading. The interviews were audio-recorded.

### Data Analysis

All interviews were transcribed verbatim by a third party. Data were analyzed following interpretative phenomenological analysis, a thoroughly described method situating participants in their specific context, exploring their personal perspectives.^15^ This method consists of 6 steps; steps 1 to 5 were independently performed by the data analysts (R.v.O. and N.K.), and step 6 was performed by all authors.

1) Reading and re-reading a transcript. For this step, the transcript of 1 interview was read, and the recording was listened to simultaneously.
2) Initial noting. In this step, the interview was coded line by line, looking specifically for experiential claims, concerns, and understandings of the case’s lived experiences.
3) Developing emergent themes. The initial codes from step 2 were turned into themes through interpretation by the data analysts. The analysts looked for the essences of what participants said.
4) Searching for connections across emergent themes. The data analysts fit together the themes and selected the themes relevant to the research question. Connections between themes were noted in 1 case description per case.
5) Moving to the next case. Steps 1 to 4 were repeated for each case.
6) Looking for patterns across cases. Patterns across cases were searched in 2 consensus meetings using the transcripts (step 1) and case descriptions (step 4).

The 2 analysts presented and explained their case descriptions. All authors looked for similarities and differences between the 2 case descriptions. After 3 unique cases, they hypothesized patterns across cases by looking for connections, potential themes, certain orders, and key emergent themes. Finally, the first author (R.v.O.) examined the individual case descriptions once more to check whether they were adequately reflected by the established themes. An example illustrating the analytic process can be found in Supplementary Material 3.

### Techniques to Enhance Trustworthiness

The transcripts were aggregated and analyzed using ATLAS.ti software (ATLAS.ti version 8.4, Scientific Software Development GmbH, Berlin, Germany) supporting open and transparent data analysis. An audit trail was used to continually check the interviewers’ interpretations with the original data in an iterative process of analysis. Furthermore, credibility and trustworthiness of interviews were enhanced by the timing of the data collection. There was a maximum of 3 months between the lived experiences in the work situation and the interview. Two different researchers independently collected and analyzed data to maximize credibility and authenticity. By using a heterogeneous sample of 4 different types of rehabilitation professionals, we created an interprofessional understanding of lived experiences. Moreover, the consensus meetings with the multi-disciplinary research team improved transparency and thoroughness of the data analysis. Transferability was improved by the provision of thick description of results, including vignette quoting. All participant quotes were translated by a third-party native English speaker.

### Role of the Funding Source

The funders played no role in the design, conduct, or reporting of this study.

## Results

### Participants

Forty-two potential participants were contacted, 3 of whom did not respond to the study invitation. In total, 39 rehabilitation professionals enrolled in the study and completed the interview. The sample consisted of 9 dieticians, 7 occupational therapists, 13 physical therapists, and 10 speech therapists. Two of the 39 interviews were held through a phone call with audio recording without video connection because of technical failure. Interviews had a mean duration of 48 minutes (SD = 6 min). Participant characteristics are displayed in Table 1.

**Table 1:** Participant Characteristics

**Participant Characteristics (N = 39)**	
Sex	
Female	29 (74%)
Male	10 (26%)
Age, y	
Mean (SD)	39 (11)
Range	24–64
Profession	
Dietician	9 (23%)
Occupational therapist	7 (18%)
Physical therapist	13 (33%)
Speech-language therapist	10 (26%)
Work experience in the hospital (in y)	
Mean (SD)	15 (11)
Range	1–42
Type of hospital	
University hospital	22 (56%)
General hospital	17 (44%)

### Theme Descriptions

Four themes emerged from the data analysis: a disease with great impact, personal health and safety, staying human in chaotic times, and solidarity and changing professional roles. Thorough descriptions of the themes are provided below to elucidate their meaning. Participant quotes are used to exemplify particulars of the phenomenon. They are numbered in correspondence to the numbers in the open access database.^21^ The theme descriptions contain ethical issues as experienced by the participants. Themes, ethical issues, and accompanying values at stake are displayed in Table 2.

**Table 2:** Themes, Ethical Issues, and Accompanying Values at Stake

**Themes**	**Ethical Questions**	**Values and Principles at Stake**
A disease with great impact	How ought I treat patients without sufficient knowledge of the disease?	Beneficence, non-maleficence, professionalism
Personal health and safety	Is it safe to work in the hospital? Am I putting my loved ones at risk?	Patient health, personal health, responsibility
Staying human in chaotic times	How do I keep a healthy work/life balance? How do I stay human in these conditions? How can I treat patients in a humane way?	Personal health, duty, humanity, dignity
Solidarity and changing professional roles	What role should I take on? How can I support my colleagues? Am I a general health care professional or a specialized hospital-based rehabilitation professional?	Responsibility, solidarity, competence

### A Disease With Great Impact

One of the first encounters of Dutch rehabilitation professionals with COVID-19 was media footage of Italian ICUs with many patients mechanically ventilated in prone position. At the end of February 2020, the first case of COVID-19 in the Netherlands was confirmed. Participants pointed out that they knew many very sick patients could come their way and heavily impact their work, which made them apprehensive. In some cases, rehabilitation professionals were trained and briefed to be able to help nurses. Non-acute care was scaled down, and rehabilitation professionals working in outpatient clinics had to call their patients to cancel appointments. Some participants experienced some very quiet shifts in which they were waiting for patients to arrive. They sensed a quiet before the storm and a looming threat (Tab. 3, Quote 1).

**Table 3:** Quotes Related to “A Disease With Great Impact”

**Quote 1**	“That was surreal. I came in, and it was actually very quiet from eight to ten and then between ten and twelve we suddenly had emergency admissions and scheduled admissions and that’s when you saw the nurses panic, because the nursing team had to be completely reorganized as they suddenly had 6 patients instead of 3.” (participant 10,320)
Quote 2	“During one of my shifts at the ICU a helicopter landed on the front lawn 3 times to take patients from the ICU to another hospital because we were full. It was also quite intense to see a long line of ambulances loading and unloading people.” (participant 36,412)
Quote 3	“One of the most intense moments [I experienced] took place on that first Friday when a man my own age was being intubated. I was born in the year ‘79 so when I heard him come in and I heard the staff say that there would soon be a man admitted that was born in ‘79, I thought, oof, he is my age. [...] In the end he did make it after 3 days. He was also off the ventilator after 3 days. I checked up on him for a while. Some people get under your skin a little.” (participant 10,320)
Quote 4	“What really got to me is seeing patients panic, but not really being able to reassure those patients, to say don’t worry, it will all work out, we know what we are doing. When it comes to other patients in the hospital you often know the course [of illness] so you can also assume a more reassuring role. So I thought that was, well, I found it quite strenuous mentally.” (participant 38,684)
Quote 5	“At one point it was like that for a few weeks and then I thought well, I would actually like to be more involved because I am getting afraid here at home, because I am not there (in the hospital). Because at home it all seems very spooky, but when you are there it is all very different.” (participant 84,710)
Quote 6	“Once you are passed the sluice room, and you arrive at the department itself, well, that felt strange, but the kind of distress or madness that you see on television and in the media, was not present at all.” (participant 33,394)
Quote 7	“It felt like you were about to be fired. Not that I was afraid of my dismissal, but it felt very weird. Because you sort of say goodbye to your colleagues and you do not know when you will see each other again. So, we ended up calling each other every day. We did plan a sort of meeting at the end of each working day and eventually it turned out that this was not necessary, instead we often connected by app and by mail. But it felt very strange.” (participant 84,710)

When the number of hospitalized patients increased, participants reported that things changed very quickly. Hospital wards were restructured to provide cohort treatment, and nursing staff was shuffled around. When hospitals reached their patient ward limits, patients were transferred to other hospitals. Participants felt like they were in a bad movie. There was a sense of disbelief and being taken by surprise (Tab. 3, Quote 2).

The rehabilitation professionals talked about a crisis mode and, among nurses and physicians, a survival mode. There was uncertainty and questions such as: What is happening? Will we be able to cope with all these patients? Participants explained that they felt anxious, especially those who witnessed severe illness due to COVID-19 among loved ones, or rehabilitation professionals living in areas with a large number of patients with COVID-19. The streets were empty because of the government measures, which made going to their work weird and surreal. Hospitalized patients were very sick, requiring high doses of oxygen; many patients were mechanically ventilated in prone position, and many patients died. The participants were overwhelmed by the severity of the illness. Some patients had great impact on the well-being of participants, especially when patients were of a similar age (Tab. 3, Quote 3).

The rehabilitation professionals saw patients being lonely and felt the urge to help and comfort them. This feeling was amplified by the fact that family and close relatives of patients were often forbidden from visiting as a result of infection precaution measures. Rehabilitation professionals saw patients in fear and panic and witnessed sad moments, which made them feel powerless. Concerning the treatment of patients with COVID-19, there was uncertainty about what to do. Rehabilitation professionals had little information about the disease. Participants did not dare to rely on their clinical experience because the disease showed an unpredictable course, very different from normal. They faced ethical issues, including how to treat patients without sufficient knowledge of the disease and its unpredictable course. Rehabilitation professionals tried to solve these ethical issues by being more careful and by including more checks and balances. Some participants felt they were failing the patients because they did not have the expert knowledge needed to effectively treat them. The participants wondered if patients would ever recover and what would be left of their quality of life (Tab. 3, Quote 4).

The participants experienced a sharp contrast between being inside and outside of the hospital. From the outside, the situation seemed eerie due to the stories being told in the media. Being inside the hospital put things into perspective according to rehabilitation professionals. There was a feeling of dread, a feeling of pressure, but at the same an excitement before and while entering the wards. Participants typically mentioned they were curious and wondering: What is happening behind those closed doors? This made it even more rousing to go through the doors or sluices (Tab. 3, Quote 5).

The participants explained that they stepped into a different world when they entered the cohort wards or ICUs. The PPE measures impacted the look and feel of the wards, creating an unpleasant personal distance between patients and professionals and causing them to lose their “human touch.” In a short time, people were used to the situation inside. It was relatively quiet in contrast to the chaotic context pictured in the media. The participants said they saw “with their own eyes that patients were still humans, irrespective of their condition.” Despite the isolation precautions, it was the scenery and work with which they were familiar. The allied health professionals noticed a difference between colleagues that had seen the wards from the inside and those who had not. According to the participants, the anxiety and feeling of threat were lower among those who had been on the inside. They wished that the other colleagues would be allowed to see the patients and wards with their own eyes, because it would put things in perspective (Tab. 3, Quote 6).

Some participants suddenly had to work from home, a change that was very abrupt and felt strange. The participants expressed feelings of emptiness, resignation, and guilt towards colleagues working in the hospital. Participants pointed out that it felt safe to be able to work from home; however, on the other hand it was disappointing that they could not take part in the clinical work. The possibilities to work remotely were seen as positive and offered good solutions. However, participants typically missed their connection with patients, certainly when they had to work with “patients from paper” (Tab. 3, Quote 7).

### Personal Health and Safety

During the first COVID-19 wave in the Netherlands, there was little information about the seriousness of the disease. There were stories that approximately 25% of the hospitalized patients would die. There were cases of health care professionals who got sick and were admitted to their own ICU. Furthermore, there was uncertainty about the way the virus spread. Participants pointed out that they were very aware of the danger of the virus, and that they took action to avoid becoming infected. They felt a great responsibility for the health and well-being of their loved ones. Participants faced the question: I want to help in the hospital, but is it safe? If I do, will I put my loved ones in danger? This made participants meticulous regarding their hygiene. Suits were changed more often, hands were washed more often, keyboards were cleaned more regularly, and they hugged their children only after first taking a shower. Some participants chose to be in very strict self-isolation to solve this issue and stayed available for work in the hospital. Participants who had to work in the hospital while they had expressed concerns to their supervisors about being infected with the virus felt guilt towards patients and colleagues who they might have exposed, which made them feel angry and frustrated (Tab. 4, Quote 1).

**Table 4:** Quotes Related to “Personal Health and Safety”

**Quote 1**	“My wife is a little older than I am and she has some lung problems, so she might be susceptible, and I didn’t want to infect her. I know that at my age I am not a big risk to my kids, but the situation I am in means that I can catch it more easily because I am exposed all day, if I take proper hygiene measures, this also applies to my grandchildren and my parents. So yes, I consciously said that I am going to go into quarantine and stay 1.5 meters away from others at home and that is quite easy to do, but you have to be aware of it and we were [.…] I didn’t want to be the one to spread it, to infect vulnerable people and loved ones so that was my main motive.” (participant 51,388)
Quote 2	“At one point I really felt much safer here in the hospital than in the supermarket. I was often asked if I was not afraid, and about wearing a suit, and how it must be quite stressful, but to be honest, I would rather be in a suit than in the supermarket.” (participant 37,470)
Quote 3	“Then I agreed with my boyfriend not to drink alcohol anymore because we felt like we wanted to stay healthy, we both work with COVID-19 patients, so we stopped [drinking]. And we started exercising, 3 times a week, together with our colleagues. That worked out very well, because it stopped us from only talking about COVID-19 at work, and changed the subject to sports. Never in my life have I exercised so much, ate that healthy, and abstained from any alcohol. [...] so strangely enough I came out fitter than I went in. No COVID-19 pounds.” [participant laughing] (participant 30,952)
Quote 4	“For me it really is like I told you, about having been ill, and at that time, it was questioned whether I could ever lead a normal life. And that took about 2 years. And then I slowly started living a normal life again. But I never dared to dream that I could do this. And now I’ve done it, so now I feel like I can take on the world.” (participant 13,293)

In addition, close relatives of participants also raised concerns about the safety of their work. They asked whether participants formed a risk because they were in contact with infected persons and asked if the participants did not feel scared to take on face-to-face contact with patients with COVID-19. Overall, participants felt safe using the PPE in the cohort wards and ICUs. Some stated that they were aware of the relatively luxurious position compared with the dearth of PPE in the primary care setting (Tab. 4, Quote 2).

Some situations caused participants to worry that they had been exposed to the virus, in particular when they witnessed disconnection of mechanical ventilation, when they questioned whether they had pressed the nose pads of the mask sufficiently, or when their skin was not fully covered by the PPE. One participant stated that she wore extra high socks to avoid skin contact. Some participants knew themselves to be hypochondriac and recognized anxiety about becoming infected as a continuing threat. Many participants opted for a healthy lifestyle to be more resilient to the virus and stay available for work in the hospital (Tab. 4, Quote 3).

The rehabilitation professionals felt anger towards people not following the rules or not keeping the required social distance. This anger was aimed at people outside the hospital, for instance in the supermarket, and also at colleagues in the hospital. Participants feeling vulnerable were extra keen regarding the measures and felt troubled when colleagues were not as compliant; however, they did not want to be the one to keep correcting them. Other participants feeling vulnerable due to previous illness considered it as a personal victory that they were able to keep working during COVID. This made professionals feel strong and energetic (Tab. 4, Quote 4).

### Staying Human in Chaotic Times

In a short period of time, adjustments were made in the hospitals, including restructuring departments for cohort, replacing staff, and implementing new treatment guidelines and protocols. Participants felt a need for clarity and leadership. Some participants recognized aspects from previous epidemics, such as coping with patients with HIV. The restructuring came with a continuous stream of information. There were many newsletters, emails, and webinars to update the health care professionals. Participants pointed out that frequent changes in policy made the situation chaotic, and they spoke of excessive information through all different channels. It was difficult to separate main from side issues. In addition, it took much time to distil what information was relevant for their work (Tab. 5, Quote 1).

**Table 5:** Quotes Related to “Staying Human in Chaotic Times”

**Quote 1**	“You don’t dare not to read it because you might miss something you need to know. So, I always read everything but, in the end, I thought hmm, does this actually make a difference for me?” (participant 58,414)
Quote 2	“Usually the door is open so you walk in and start treating someone and you occasionally do consult with the nursing staff of course, but that happened more often now because the door is closed and you don’t really want to waste materials [PPE], so you ask in advance, who’s behind the door? How are they doing? What should we do? What do we want to do? We need to make choices. So, we actually added a step [to the process] in order to choose what is necessary and what is not, and where I should go” (participant 58,247)
Quote 3	“I also quite liked the fact that a lot of meetings that you usually had to attend had been canceled. So, it certainly has advantages. So yes, I do hope we can hold onto some things from this period of time.” (participant 26,976)
Quote 4	“What really, well, what really affected me was the 1 nurse who addressed people by name and told them [what we were doing] we will now do this or do that. I really liked that, so I picked it up and started doing it myself. I started addressing people too. Otherwise, it feels like a rag doll lying there.” (participant 58,414)
Quote 5	“People also feel really lonely don’t they, and as I said, all doors are closed, you have to rely on that one moment when someone comes in, all suited up, well then, that real contact, that real human contact is not there really and it is that human interest that I think we need to make sure we hold on to.” (participant 66,521)

Rules and policies changed repeatedly. The rehabilitation professionals experienced this period as chaotic and hectic; they were required to be alert all day. This also concerned PPE usage. Patients required care in full isolation, and at the same time scarcity of PPE material resulted in orders to avoid patient contact unless strictly necessary. Rehabilitation professionals felt pressure to not use any materials unless it was very urgent. Working with the PPE was physically challenging: participants mentioned they felt dehydrated, and experienced headaches in the evening. They had to be creative in unfamiliar circumstances, avoid entering and exiting rooms and departments, and find solutions in the use of materials because the normal instruments were not available at cohort wards and ICUs or could not be used due to limited options for cleaning. Moreover, they had to work with inexperienced teams and colleagues because nurses were oftentimes relocated. With many factors different, participants were happy to recognize their colleagues on the work floor. Working in this new situation and with this new disease, participants explained that they could no longer rely on their routine, which made their workdays intense and exhausting (Tab. 5, Quote 2).

The period was experienced as intense and stressful, but on the other hand also valuable. After work they might feel tired, irritable, and hot-tempered. Some participants experienced difficulty sleeping. They took their work home and had to process their experiences. Participants were confronted with the question: I want to fulfill my job in the best possible way, but how do I keep a healthy work/life balance? It helped to share stories and feelings with loved ones. Others explained that they had to write things down or take time to ponder to process things. After the COVID period, they felt tired and needed a period to relax. Participants experienced the workload in different ways. Those with small children explained that it was a very busy period due to the home situation with home schooling. Others stated that the period was quite relaxed because a lot of meetings were cancelled and appointments with friends and family were cancelled (Tab. 5, Quote 3).

The unfamiliar chaotic situations, with all professionals dressed in PPE and with many sick, often sedated, patients with COVID-19, made the work at the wards and the ICUs feel less humane than normal. During the crisis, there was high turnover, and many patients died. Therefore, some participants felt that they blocked their emotions. They experienced a “robot mode,” and the work felt factory-like. This typically was the case for rehabilitation professionals participating in teams that supported ICU nurses turning patients in prone position. Participants felt that they were treating bodies, or human-like dolls, rather than people. Some situations were referred to as disgraceful. Patients were oftentimes naked, smelly, bloated, and affected by severe decubitus, which rehabilitation professionals had not experienced before. There was a lack of personal information in the rooms, which made it impossible to provide person-centered care. This felt wrong, and some participants felt guilt towards those patients. They wondered: how do I stay human in these conditions? And how can I treat patients in a humane way? They coped with this by talking to patients and by showing dignity and respect, even when patients were deeply sedated and were not expected to hear anything of the things said (Tab. 5, Quote 4).

The rehabilitation professionals wanted to restore and promote the patients’ dignity as much as possible. Some participants explained that they worked in health care because they wanted to care for people. They felt the need for human contact and empathized with the lack of contact patients received (Tab. 5, Quote 5).

The participants stressed the importance of a personal connection when motivating patients. Participants found creative ways to establish human contact despite the PPE boundaries. They sought eye contact more consciously and frequently or used their voice in particular ways. Some participants explained that they very deliberately showed their face to the patient before entering the room.

### Solidarity and Changing Professional Roles

The period of crisis was experienced as a time of solidarity. Participants typically felt an urge to support their colleagues, to relieve busy physicians and nurses, and to help keep the hospital up and running. Participants speak of a caring reflex, a duty, a calling, a feeling of commitment, a need to stand by when needed, to be of service. They felt drawn to the hospital. They felt that this was why they had become a health care professional. Many hospital-based rehabilitation professionals were deployed as general health care professionals. Some helped transport patients through the hospital, and others were stationed at the nursing ward to support nursing staff. They helped with washing, replenishing stocks, distributing medication, and so on (Tab. 6, Quote 1).

**Table 6:** Quotes Related to “Solidarity and Changing Professional Roles”

**Quote 1**	“On my first day off, I went cycling. That is my hobby, so I went for a leisurely ride, reducing stress. And I as cycled towards [city name], I was inclined to ride to the hospital. At that moment you realise that you are constantly taking on the assisting role. It didn’t matter what you did, either. I even bathed a patient; those are nursing duties. The nurse asked me if I wanted to bathe the patient, and I said I never did that before but—by then I’d also seen a lot of feces, and at that point that doesn’t matter as long as you can help the nurses. They are very busy.” (participant 10,320)
Quote 2	“You just do it. In my opinion the risk you took was small compared with the gains of work satisfaction. So, you help the more vulnerable, the very sick, and that feels like a small investment you can make in order to contribute to society. I was very proud of my work at that time.” (participant 96,116)
Quote 3	“In matters of life and death, what should an occupational therapist come around for? It was like, family became very important, as did social work and spiritual guidance, they are very important. It was very medical and then, well, within the entire ICU, at that level of care, there is nothing for you to do as an occupational therapist.” (participant 48,827)
Quote 4	“On the ICU you usually take the lead as a physiotherapist and the nurse has a supportive role and now, we had to, well, search for a new division of roles in which the nurse actually wanted to take the lead. I noticed, especially in that first week, that the nurses were really taken aback, and it took a bit of getting used to, like oh, the tube might come off and no, you can’t touch anything. […] And as you are a physiotherapist at heart, you will not simply turn the patient over, but you also look at the overall mobility to get a bit of a feeling. Because you don’t know that [patient] population either.” (participant 40,447)
Quote 5	“The theme leader nutritionist discussed with a privacy officer whether we could also view the patients’ files without referral. And because it was to the benefit of the patient’s treatment, I think we actually received approval for it that same day. When usually you have to get approval from 3 committees, so to speak, it was now arranged within a day. So that is something that changed with Covid-19, now things could be arranged more quickly.” (participant 83,109)
Quote 6	“Now we are back to normal and I actually had that feeling already after 3 weeks at the ICU when I thought, ah, okay, we have returned to our regular hierarchy.” (participant 40,447)

Some participants stated they felt frustration when they were not allowed to help on the wards due to rules or management choices. When coming to and working on the wards or ICUs, they felt welcomed and appreciated. There was a strong sense of team spirit among health care workers. The participants felt proud that they were able to fulfill their role (Tab. 6, Quote 2).

From outside the hospital, participants also felt gratitude and respect in the form of banners, messages, fruit baskets, and gifts sent to the wards. There were 2 negative cases in this theme: 2 participants explained that they did not see any role for themselves. They felt that, in a time of life and death, rehabilitation professionals should exercise restraint and not place themselves in the foreground (Tab. 6, Quote 3).

Other rehabilitation professionals helped turn patients in the ICUs because there were many patients being mechanically ventilated in prone position. They were staffed to assist nurses, whereas they are accustomed to being in lead positions. Some participants explained that this was strange and confusing. It was not clear what was expected from them and what role they should take. They questioned: What role should I take? Rehabilitation professionals also had questions regarding their identity, for example: “Am I a general health care professional or a specialized rehabilitation professional?” As a general health care professional, they particularly wanted to support nurses and physicians to reduce their workload. As a rehabilitation professional, they critically considered what care ought to be provided (Tab. 6, Quote 4).

Sometimes, based on their skill and knowledge, rehabilitation professionals saw room for improvement in the situation; however, they were not sure whether feedback was appreciated in this situation. They were aware of the workload among the nurses and did not want to be an additional burden. Some participants ensured work would not be delegated to nurses on the floor. On the other hand, participants had difficulty not perceiving the situation through their allied health lenses. The new disease also raised some professional curiosity. They wanted to add their knowledge and skills where they thought they could be beneficial to the patient. They felt pride in their profession and sought recognition for it. Several participants pointed out that they felt capable of taking a role as an expert during the COVID-19 crisis. They took this opportunity to show health care professionals in other disciplines what they were capable of. For instance, the dieticians helped determine the food demand of patients in the ICU to relieve ICU physicians from this task. It felt like a victory when this was established and appreciated. In several different ways, participants felt room to adopt roles they were normally not able to. Some were national experts and taught webinars. Others experienced that they could finally have substantive talks with physicians. The hierarchy seemed to vanish. Moreover, during the crisis period, many factors could be organized within a short time. These elements together made the period energizing, exciting, and instructive (Tab. 6, Quote 5).

It was curious to note all the possibilities and opportunities in this period, which made possible the accomplishment of tasks that had long been desired. This felt refreshing and provided an energy boost. Participants wished they could maintain aspects of this new atmosphere. After the crisis period, participants felt recognition and goodwill on the wards. However, some participants pointed out that the hierarchy and bureaucratic procedures started to return, which felt frustrating (Tab. 6, Quote 6).

## Discussion

This qualitative study using interpretative phenomenological analysis to explore the lived experiences of rehabilitation professionals working in a hospital during the COVID-19 pandemic revealed 4 themes: disease with great impact, personal health and safety, staying human in chaotic times, and solidarity and changing professional roles. Rehabilitation professionals felt welcomed and appreciated at the COVID-19 wards and ICUs and were proud that they were able to fulfill their roles. The themes and accompanying ethical issues reflect a wide range of situations that were morally complex and led to moral distress.

Participating rehabilitation professionals indicated that, during the first wave of the COVID-19 crisis in the Netherlands, the virus had a great impact on the in-hospital working environment due to the massive downscaling of regular care, infection prevention measures, and unknown risks to rehabilitation professionals’ personal health. Normal structures, frameworks, protocols, agreements, roles, and certainties did not meet the crisis requirements. This was frightening for the rehabilitation professionals and was accompanied by a need for structure and leadership. New work structures and guidelines were developed, which generated excessive information. The rehabilitation professionals had difficulty to distinguish main from side issues and to distil what information was actually relevant for their work. At the same time, participants experienced a certain freedom, which made room for authentic motives, connection, and solidarity. In the chaotic situations, rehabilitation professionals were urged to rely on their intuition and started acting accordingly. Rehabilitation professionals felt a calling, experienced a sense of being needed, and felt which parts of their work were particularly meaningful, such as therapy for patients and support of nurses. These aspects were pointed to as being beautiful, inspiring, and providing energy. Organizational changes could be arranged within a short time, including changes otherwise impossible to achieve under normal circumstances. Rehabilitation professionals were keen on sharing their expertise and were professionally interested to treat this new patient group as well as possible. Despite the difficult conditions and isolation precautions, they sought ways to remain human in line with their fundamental attitudes to care for people and engage in human interactions. Rehabilitation professionals hoped that these meaningful changes from the crisis period would remain. However, they recognized that when the crisis waned, previous hierarchy and bureaucratic procedures seemed to reappear.

Our findings are in line with the experiences of 22 professionals caring for patients with COVID-19, so-called “soldiers on the front,” between May and August 2020.^12^ Rehabilitation professionals have also been working on the so-called front line and discuss the same challenges, such as balancing between “being part of something bigger” (in this study: being needed) and “putting family at risk” (in this study: personal health and safety). Our findings are also consistent with other studies reporting experiences of health care professionals treating patients during a pandemic.^11^ This makes it clear to us that past lessons learned contribute to current health care and that lessons learned now contribute to better health care in the future.

In the participant experiences in this study, several types of moral distress as described by Morley et al can be recognized.^22^ Moral dilemma distress was present when allied health professionals experienced the dilemma that they wanted to help nurses and physicians but did not know whether it was safe to work in the hospital, not wanting to put their loved ones at home at risk. Moral values and principles such as professional loyalty and personal health were at stake, because participating in health care would be loyal, though it could lead to viral spread to loved ones. Moral uncertainty distress was present in the lack of knowledge of the disease, expressed in the values competence, beneficence, and non-maleficence relating to rehabilitation professionals’ insecurity about how to treat patients in the best way; they did not dare to trust their clinical experience. Moral constraint distress occurred in relation to values such as professionalism, responsibility, and duty, for instance, when rehabilitation professionals were not able to treat patients due to isolation restrictions and shortages in PPE material.

Moral distress is a natural response to morally difficult encounters in the provision of patient care.^22^ Ethical conflicts and moral distress will always exist in health care, especially in high-intensity settings with ethical decision-making.^23^ To avoid the long-lasting negative impact of moral distress, efforts should be made to mitigate its effects. Until now, only a few evidence-based interventions have been studied, with limited effectiveness.^24^ Yet, some promising practices can be suggested.^22^^,^^23^^,^^25^ The recently developed SUPPORT model enables organizations to simultaneously develop ethical skills and facilitate team-based dialogue while creating policies shaped by standards of healthy work environments.^23^ Based on this model, ethical issues should be recognized and acknowledged. Ethical dialogue should become normal practice, and safety should be established for discussions among team members. The organization should encourage debriefing and create conditions to engage in ethical reflection. The importance of ethical reflection is also stressed in Kunneman’s concept of normative professionalism.^26^ It enables professionals to create room and reflect on “slow questions.”^27^ Slow questions concern life questions about relations, health, loss, violence or longings, issues that cannot be solved by quick technological solutions. The ethical issues faced by rehabilitation professionals in the COVID-19 crisis were morally complex, requiring a pragmatic tradeoff of values in the search for good care and therefore requiring recognition and acknowledgment.

Limitations of this study include the lack of data triangulation. The use of other data collection methods, for instance participatory observations, could have enriched the data. However, due to the limited availability of PPE and the risk of virus transmission, this was not an option. Because of the risk of viral spreading, interviews had to be performed through video calls. Video calling might have limited the richness of the interviews because of less rapport between interviewer and participant and less non-verbal communication. However, recent studies suggest that in-person interviews are only marginally superior to video calls.^28^^,^^29^ No test interviews were performed before the start of data collection, which might have influenced the data collection during the first couple of interviews. However, because all authors had been involved in caring for patients hospitalized with COVID-19, they possessed a clear vision on the topic, and, over time, the interview guide was only marginally adjusted. For this study, we interviewed rehabilitation professionals in 4 different disciplines, creating an interprofessional understanding of lived experiences. However, only the perspective of employees was sought, whereas the perspectives of managers of organizations, or politicians countrywide might be opposing. All study participants were rehabilitation professionals employed in Dutch hospitals. The generalizability of findings might be limited because rehabilitation professionals in other countries might have been working under different circumstances. Furthermore, the severity of the COVID-19 crisis differed across countries. However, the curve of hospitalizations was similar to other countries, at least in Western Europe. Furthermore, with the aid of thick description of the results, readers can decide for themselves to what extent our findings are transferable to the reader’s situation.

In conclusion, during the COVID-19 crisis, rehabilitation professionals faced a wide range of situations that were morally complex and led to moral distress, requiring a pragmatic tradeoff of values in the search for good care and therefore requiring recognition and acknowledgement. To diminish the long-lasting negative impact of the COVID-19 pandemic and moral distress, employers should empathize with the experiences of hospital-based rehabilitation professionals and create conditions for ethical reflection. Our data underline that hospital-based rehabilitation professionals value professional autonomy; hence, creating room for professional autonomy helps them feel needed, connected, and energized. However, the needs of hospital-based rehabilitation professionals may conflict with organizational rules and structures.

## Supplementary Material

ptj-2021-0860_r2_suppl_material_1_pzac052

PTJ-2021-0860_R2_Suppl_Material_2_pzac052

PTJ-2021-0860_R2_Suppl_Material_3_pzac052

## Data Availability

Data are digitally secured at the Radboudumc and available upon reasonable request.
