# Supplementary material for: A Sense of Being Needed: A Phenomenological Analysis of Hospital-Based Rehabilitation Professionals’ Experiences During the COVID-19 Pandemic
Source: Phys Ther. 2022 May 5;102(6):pzac052. doi: 10.1093/ptj/pzac052 (PMC9129175; doi:10.1093/ptj/pzac052)
Supplement: PTJ-2021-0860_R2_Suppl_Material_2_pzac052 [file ptj-2021-0860_r2_suppl_material_2_pzac052.pdf]

Supplementary Material 2 - Interview guide

Introduction and start recording	I would like to ask you to answer as comprehensively as possible during the interview. This gives me a picture of your experiences and the meaning you give to them.
Working in the hospital	Can you tell me about your work in the hospital as a dietician/occupational therapist/physical therapist/speech-language therapist during the COVID-19 crisis?<br><br>Prompt: Can you describe a working day? / How was it to work in the hospital? / How was it to be in the hospital?
Working at the COVID-19 ward	How was it to work at the COVID-19 ward?<br><br>Prompt: Differences to other wards during that time? Differences to normal times? Example? Impressions? Things you missed? Things you could not do? Difficult decisions?
Working at the COVID-19 ICU, when relevant	Did you work at the COVID-19 ICU? How was it to work at the COVID-19 ICU?<br><br>Prompt: Differences to the wards during that time? Differences to normal times? Example? Impressions? Things you missed? Things you could not do? Difficult decisions?
Screening, evaluating and testing	How did you screen, evaluate and test patients with COVID-19?<br><br>Prompt: What actions did you take? Examples? Difficulties? Changes over time?
Treatment	How did you treat patients with COVID-19?<br><br>Prompt: What treatments? Examples? Difficulties? Changes over time?
Ending treatment and arranging hand-over	How did you end your treatment and arrange a hand-over?<br><br>Prompt: Which patients received after-care? What was your role? Difficulties? Changes over time?
Personal protection equipment	How was it to work with personal protection equipment?<br><br>Prompt: Differences to normal? Changes over time? Communication difficulties?
Ethical issues	Can you tell me about possible difficult situations or difficult choices that you had to make during your work?<br><br>Prompt: Example? Causes? Solutions? How did you feel in these situations? How did you deal with these situations?
Health	How did you experience your own health before and during the crisis?<br><br>Prompt: Fitness? Insecurities? Work load? Changes over time?
Participant characteristics	What is your gender and age?<br><br>How many years of experience do you have in this profession?

Closing and stop recording	Would you like to say something in general about your experiences as a rehabilitation professional with patients with COVID-19? Is there a topic or event that has not yet been addressed during the interview?
