# Supplementary material for: A Sense of Being Needed: A Phenomenological Analysis of Hospital-Based Rehabilitation Professionals’ Experiences During the COVID-19 Pandemic
Source: Phys Ther. 2022 May 5;102(6):pzac052. doi: 10.1093/ptj/pzac052 (PMC9129175; doi:10.1093/ptj/pzac052)
Supplement: PTJ-2021-0860_R2_Suppl_Material_3_pzac052 [file ptj-2021-0860_r2_suppl_material_3_pzac052.pdf]

Supplementary Material 3 - Example of thematic process

**Initial noting**	**Developing emergent theme**	**Case description (fragment)**	**Key emergent theme**
We were overloaded by the many sick people despite the warnings from Italy and China.	Being overloaded by the number of sick patients.	This physical therapist worked at the intensive care unit. The virus heavily hit the region and they were overloaded by the large amount of sick patients. It was bizarre to see the department full of severely ill and mechanically ventilated patients, oftentimes in prone position. At some point the hospital almost only treated patients with COVID-19.	A disease with great impact
The amount of newly admitted patients and dying patients with COVID-19 really got to me.			
I found it very disturbing to see the ambulances line up in front of the hospital.			
In the intensive care unit we are used to very sick people, but I have never seen so many.			
The helicopter landed several times in the garden that day to transport patients to other hospitals, that was impressive.			
