# Supplementary material for: A Sense of Being Needed: A Phenomenological Analysis of Hospital-Based Rehabilitation Professionals’ Experiences During the COVID-19 Pandemic
Source: Phys Ther. 2022 May 5:pzac052. doi: 10.1093/ptj/pzac052 (PMC9129175; doi:10.1093/ptj/pzac052)
Supplement: Supplementary file 1 [file ptj-2021-0860_r2_suppl_material_1_pzac052.pdf]

Supplementary Material 1 - Operational definitions of ethical concepts

Term	Operational definition
Norms	Rules that prescribe which actions are permitted, forbidden, or required.[30]
Values	Moral values are lasting matters or convictions that people feel should be strived for in general to realize a just society or lead a good life.[30]
Ethical principles	The four principles of medical ethics, formulated by Beauchamp and Childress in 1979, are respect for autonomy, beneficence, non-maleficence, and justice.[31] These mid-level moral norms together form a framework to guide ethical judgment.
Ethical issue, ethical conflict	Ethical issues or conflicts occur when values and norms conflict, or when they no longer seem applicable. These conflicts may occur within a person, between different persons, or there may be disagreement with the rules or structures that guide this person's professional activities.4, 5
Ethical dilemma	An ethical dilemma is a particular type of ethical problem, in which there are two (or more) options for action and whatever the person chooses, a moral wrong is committed.[30]
Moral distress	Campbell et al. define moral distress as: "One or more negative self-directed emotions or attitudes that arise in response to one's perceived involvement in a situation that one perceives to be morally undesirable."[7]
